# Solidification Cracking Restraining Mechanism of Al-Cu-Mg-Zn Alloy Welds Using Cold Metal Transfer Technique

**DOI:** 10.3390/ma16020721

**Published:** 2023-01-11

**Authors:** Zhuoxin Li, Lingshan Ou, Yipeng Wang, Hong Li, Mariusz Bober, Jacek Senkara, Yu Zhang

**Affiliations:** 1 Institute of Light Alloy and Processing, Faculty of Materials and Manufacturing, Beijing University of Technology, Beijing 100124, China; 2 Department of Welding Engineering, Faculty of Production Engineering, Warsaw University of Technology, 02-524 Warsaw, Poland

**Keywords:** cold metal transfer, solidification cracking, aluminum alloys, dendrites

## Abstract

Aluminum alloy 7075 (with 7055 and 7150 filler wires) was welded using a digital welding machine that can switch arc mode between MIG, CMT and CMT+P modes. The transverse-motion weldability test of joints welded under different arc modes showed that the solidification cracking susceptibility was lower in CMT-technique-based welds than in MIG welds. The temperature cycle of the welding pool under different arc modes was recorded using mini-thermocouples, which showed that the cooling rate was lower in CMT welded samples than in MIG welded samples. The low cooling rate promoted the growth of α-Al dendrites through the back diffusion effect. Electron probe micro-analysis showed that micro-segregation of the α-Al dendrites was lower in the CMT welded samples than in the MIG welded samples. The *T-(f_Al_)*^1/2^ curve of each weld was calculated, which showed that CMT-based welding enhanced the bridging of adjacent α-Al dendrites, reducing the tendency for solidification cracking.

## 1. Introduction

Aluminum alloys are widely used in the aviation and military industries for their high specific strength [1,2,3]. However, the high Cu-Mg-Zn content in these alloys inevitably reduces their weldability, which is mainly reflected in their high susceptibility to solidification cracking [4]. During the non-equilibrium solidification process of 7xxx series aluminum alloy, the alloying elements are segregated at the inter-dendrite zone to form a low-melting-point eutectic phase, which hinders the bridging of adjacent α-Al dendrites. The generation of solidification cracks reduces the performance of the welded components [5,6]. Therefore, understanding the fundamentals of the Al-Cu-Mg-Zn aluminum alloy solidification cracking mechanism and the corresponding methods to reduce the cracking is necessary.

High-strength Al-Cu-Mg-Zn aluminum alloys are susceptible to solidification cracking owing to their high linear expansion and wide semi-solid temperature gap [7,8]. Metal inert gas welding (MIG welding) is a typical fusion welding method for aluminum alloys, and it is widely used due to its high productivity [9,10]. In common MIG welding mode, Al-Cu-Mg-Zn aluminum alloy welds exhibit large deformation and high solidification cracking susceptibility. Cold metal transfer welding (CMT welding) is a kind of low-heat-input welding method that possesses special arc modes. During the short-circuit phase in a globular transfer cycle in CMT welding, the current drops to near zero, retracting the wire and dramatically decreasing the heat input. Thus, the thermal deformation of the workpiece is reduced [11]. Moreover, heat is transferred from the welding pool to the wire through the droplet during the short-circuit stage [12,13]. The low-heat-input feature during the CMT welding potentially reduces the solidification cracking susceptibility of Al-Cu-Mg-Zn aluminum alloy [14,15].

During the fusion welding process of aluminum alloys, a semi-solid zone exists at the tail of the liquid pool, called the mushy zone. Within the mushy zone, α-Al dendrites grow from the solid weld metal toward the boundary of the liquid pool. Under the tension caused by solidification shrinkage, the residual liquid at the inter-dendritic zone of the mushy zone tears apart to form solidification cracks [16,17]. Li Liqiong et al. [18] reported that during plasma–MIG hybrid welding of 7075-T6 aluminum alloy, solidification cracking susceptibility decreased with a decrease in heat input. Wojciech Stopyra et al. [19] reported that reducing the content of silicon impurities in AA7075 powder could lower sensitivity to solidification cracking in laser powder bed melting. Huang et al. [20] studied the effect of wire feeding speed on the susceptibility to solidification cracking in cold metal transfer (CMT) welding of AA6061 aluminum alloy, and they found that an increase in wire feeding speed first increased the solidification cracking susceptibility of the weld and then decreased it. For the perspective of the effect of the low-heat-input fusion welding method, especially the CMT technique, on the dendritic growth and solidification cracking susceptibility of the weld, more studies on the solidification cracking phenomenon in Al-Cu-Mg-Zn aluminum alloys in CMT welds are required.

A comparative study between MIG and CMT welding of 7075 aluminum alloys (AA7075) was conducted. The solidification cracking susceptibility of each weld was analyzed using the transverse-motion weldability (TMW) test [21]. In addition, liquid–solid phase evolution (temperature vs. phase fraction curves) under different cooling rates was calculated using the phase diagram calculation technique (CALPHAD) to reveal the solidification cracking restraining mechanism in CMT welding. The solidification cracking susceptibility of an Al-Cu-Mg-Zn aluminum alloy CMT joint was characterized for the first time in this work.

## 2. Materials and Methods

### 2.1. Welding Materials

A 3 mm thick AA7075 base metal, and AA7055 and AA7150 (customized) filler wires were used in the present study (Table 1). Both bead-on-plate welding and solidification cracking susceptibility tests were performed. The welding device was the Fronius-TPS5000-CMT series digital welding machine, equipped with an RCU5000i remote controller, in which arc mode can be switched between MIG, CMT and CMT+P modes. Specifically, in CMT+P mode, several pulse currents are added in each phase, which places the heat input under precise control. The welding parameters are shown in Table 2. The welding gun was tilted 15 deg toward the joint, and the filler wires’ diameter was 1.2 mm. The bead-on-plate welding was performed on AA7075 base metal of 3 × 152 × 172 mm (with 7150 and 7055 filler wires). Each workpiece/wire combination was welded under three arc modes. The samples were observed and analyzed using the optical microstructure (OM) and electron back-scattered diffraction (EBSD) techniques. The samples were taken near the crater of each bead-on-plate welding joint. The samples for the electron probe micro-analysis (EPMA) and chemical titration test were cut from the middle of the weld.

**Figure 1:**
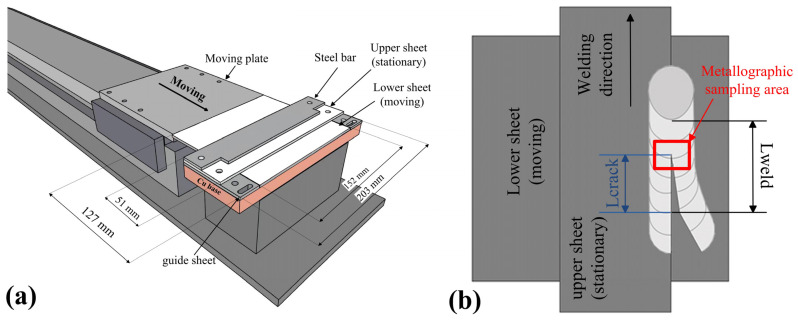
Apparatus for the present crack susceptibility test: (**a**) local dimension diagram; (**b**) calibration of sensitivity to solidification crack.

The solidification cracking susceptibility of each weld was tested using the transverse -motion weldability test (TMW test). The TMW test [21] was conducted using MIG and CMT welding on sheets of AA7075 (Figure 1a). The lower sheet was 3 mm thick, 152 mm wide and 127 mm long, whereas the upper sheet was 3 mm thick, 203 mm wide and 51 mm long. The lower plate was first pushed using a servo motor-driven slide at a speed of 90 mm/min to ensure that a significant solidification crack was created. Then, the speed was reduced to 25 mm/min to allow crack propagation. When propagation of the crack stopped, the servo motor automatically stopped. The servo motor was set at a maximum torque of 2.4 Nm (Newton meter).

During the TMW test, the pushing speed of the lower sheet was programmed into a two-level ladder pattern. The initial speed was set at 90 mm/min for initial crack formation within the weld. Then, the secondary speed was slowed to a testing variate *V* (deformation rate), which represented the solidification cracking susceptibility tendency. The value of the deformation rate *V* was switched from large to small during the test. The length of the propagation crack was labeled as *L_crack_*, whereas the length of the weld without the crack was labeled as *L_weld_* (Figure 1b). The ratio of *L_crack_/L_weld_* was the normal length of crack propagation. The data for the normal crack length plotted against the deformation rate *V* were recorded. A transition range that existed between no-cracking parameters and full-cracking parameters was reflected. The speed when the crack began to extend was defined as *V_n_*, while that when the crack was fully extended was defined as *V_f_*. The higher the critical deformation rate (*V_n_* and *V_f_*), the lower the sensitivity of the solidification crack.

### 2.2. Temperature Measurements

A high-speed photography technique was used to observe the globular transfer AA7075/7150 welding under MIG, CMT and CMT+P arc modes. A high-speed camera (i-SPEED 716) of 3000 frames per second was used to record the droplet transfer process. The temperature cycle of the welding pool was recorded using a K-type mini-thermocouple with a probe diameter of 1.75 mm. A TP700 multiplex data logger from TOPRIE was used to record the temperature data (the data acquisition interval was 100 ms).

**Figure 2:**
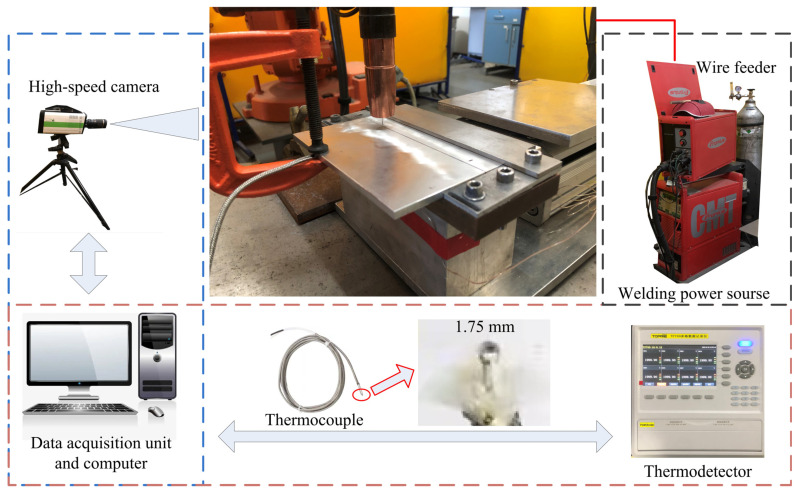
The setup of the high-speed photography and temperature measurement.

Figure 2 shows the high-speed photography and the temperature measurement process. The probe of the mini-thermocouple was plugged into the path before welding to capture the temperature cycle data of the welding pool.

### 2.3. Material Characterization

After the TMW test, the crack tip area of the sample was cut to observe the relationship between the cracks and the dendrites, as shown in Figure 1b. All the metallographic samples were etched using Keller’s reagent (5 mL HNO3, 3 mL HCl, 2 mL HF and 190 mL distilled water), and the microstructure was observed using an optical microscope (OLYMPUS BX51M).

The alloying element distribution of the welds under different arc modes was characterized using electron probe micro-analysis (EPMA, Shimadzu EPMA-1720, Kyoto, Japan). Samples were obtained from the middle zone of each bead-on-plate welding weld. Moreover, the dendritic morphology near the crater in different samples was characterized using electron back-scattered diffraction (EBSD, Zeiss Supra 55).

### 2.4. Solidification Cracking Susceptibility Analysis

This study further analyzed the solidification cracking susceptibility for each weld under different arc modes using Kou’s model. In Kou’s model, the *T-(f_S_)*^1/2^ curve was calculated to analyze the solidification cracking susceptibility [22]. The high content of the alloying elements largely creates a low-melting-point eutectic phase, generated within the AA7075 weld. This leads to deviations that reveal the solidification cracking susceptibility by using fraction solid (*f_S_*) (which includes α-Al and intermetallic compounds). Therefore, in this study, fraction α-Al (*f_Al_*) rather than *f_S_* was used to analyze the solidification cracking susceptibility.

As shown in Formula (1), three factors that affect the solidification cracking were considered: the effects of the separation of α-Al dendrites under thermal strain; the growth of dendrites toward each other; and the liquid feeding toward the channels between dendrites. The cracking criterion can be presented as (when solidification reaches the ending stage)

(1) $$\underset{}{\frac{d\varepsilon_{local}}{dt}}>\underset{}{\sqrt{1-\beta }\frac{d\sqrt{f_{Al}}}{dT}\cdot \frac{dT}{dt}}+\underset{}{\frac{d}{dz}\left[\left(1-\sqrt{1-\beta }\cdot \sqrt{f_{Al}}\right)v_{z}\right]}$$

where *ε_local_* is the local tensile strain within the mushy zone; *t* is the time; *β* is the solidification shrinkage; *f_Al_* is the fraction α-Al; *T* is the temperature; *z* is the axial direction of dendritic growth; and *v_Z_* is the velocity of liquid feeding.

The phase evolution during the non-equilibrium solidification and the welding were calculated using a commercial database, JMatPro. Both Scheil and back diffusion modes were used to analyze the effect of cooling rate on solidification cracking susceptibility. The Scheil mode assumed no diffusion within the fraction solid, which was suitable to simulate the extreme non-equilibrium solidification process. In the back diffusion mode, the diffusion within the fraction solid was considered according to the cooling rate.

## 3. Results and Discussion

### 3.1. Effect of Arc Mode on the Thermal Cycle

**Figure 3:**
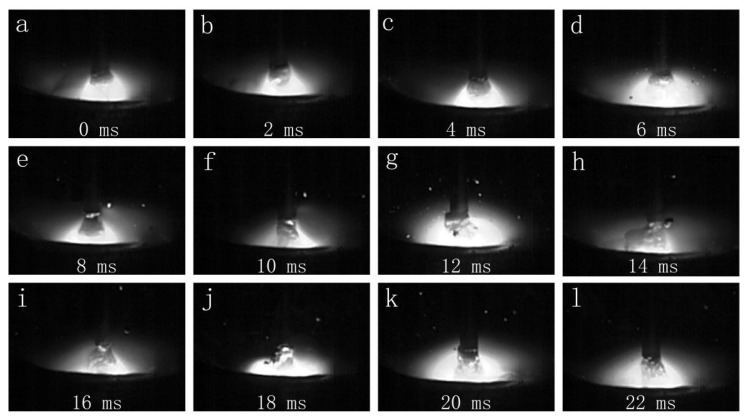
The droplet transfer process of MIG mode (7150 filler wire): (**a**–**l**) show the droplet transfer process in chronological order.

The globular transfer images under MIG arc mode are shown in Figure 3. Figure 3a–l show that the arc was continuously burning and that the molten droplets were in a drop transition. The images reveal that heat was continuously produced in MIG mode.

**Figure 4:**
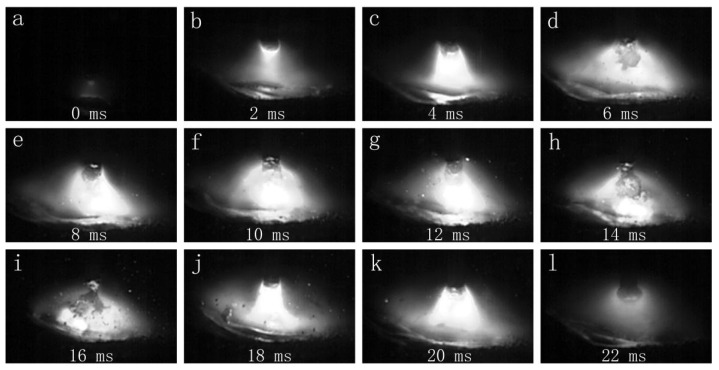
The droplet transfer process of the CMT mode (7150 filler wire): (**a**–**l**) show the droplet transfer process in chronological order.

Figure 4 shows the globular transfer images under CMT arc mode. The welding current dropped to near zero during the short-circuit stage in the CMT welding process, which led to its low-heat-input feature [23]. Compared with its counterpart under MIG arc mode, the thermal deformation was lower in the joint welded under CMT arc mode [12,13,24,25,26].

**Figure 5:**
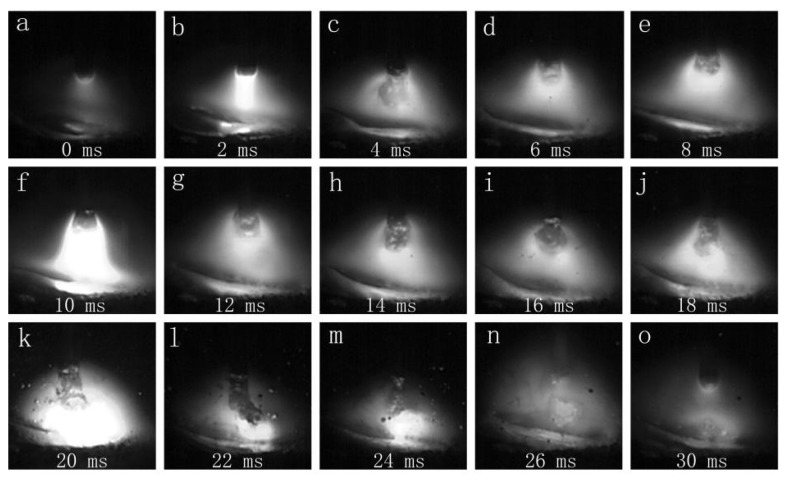
The droplet transfer process of the CMT+P mode (7150 filler wire): (**a**–**o**) show the droplet transfer process in chronological order.

Figure 5 shows the globular transfer images under CMT+P arc mode. Several current pulses were added between each arc-on and short-circuit stage in CMT+P mode. The tiny droplet formed at the wire tip during the peak pulse phase, which was transferred into the melting pool by one-pulse-one-droplet mode [27].

**Figure 6:**
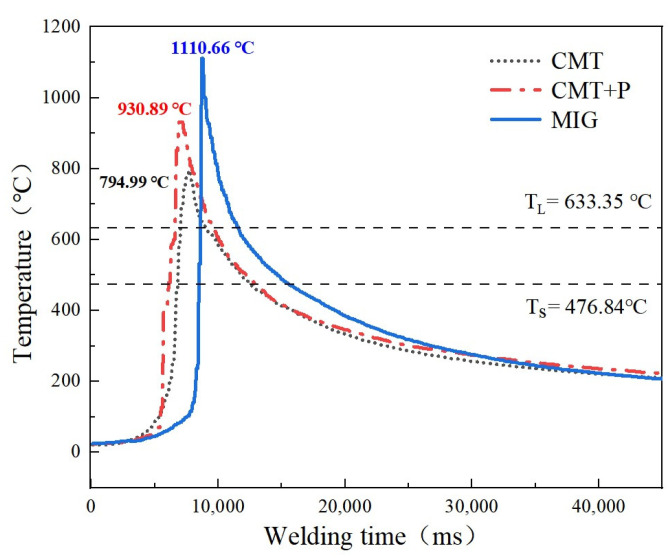
Temperature cycle of the welding pool (AA7075 workpiece with 7150 filler wire).

The temperature cycle data of the welding pool under different arc modes are shown in Figure 6. These data further demonstrate that the peak temperature and cooling rate of the welding pool in CMT or CMT+P modes were lower than their counterparts in MIG mode. Precisely, the liquidus *T_L_* and solidus *T_S_* of AA7075 were calculated using the Scheil model, which is presented as the horizontal line in Figure 6. Similar thermal cycling profiles were obtained using infrared thermography for temperature characterization during CMT welding by R. Frappierh et al. [28]. In addition, Amin S. Azar [29] developed a heat source model for CMT welding, which revealed that the droplets formed at the weld tip were transferred at relatively low temperatures (compared with typical MIG welding). This was also similar to the results of the present study (Figure 6).

### 3.2. Microstructure of the Al-Cu-Mg-Zn Welds

**Figure 7:**
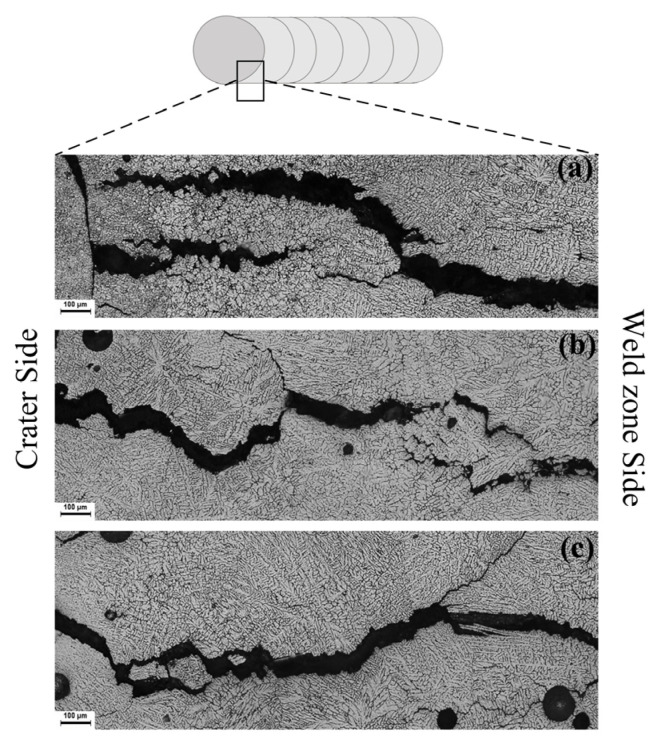
Microstructure adjacent to the crater of the weld (with 7150 filler wire): (**a**) MIG mode; (**b**) CMT mode; (**c**) CMT+P mode.

Figure 7 shows the microstructure of the crater area of the samples welded under different arc modes. Although no macro-cracks could be observed in bead-on-plate welding samples, an optical microscope revealed tiny cracks in the crater area. The arc was turned off suddenly (high cooling rate), developing a pattern of the microstructure within the mushy zone adjacent to the tail of the welding pool in the crater area. For MIG mode, it was observed that the α-Al dendrites were fine with under-developed features (Figure 7a). In comparison, the α-Al dendrites were ripened under CMT and CMT+P modes. The ripening effect in the solidification process refers to the gradual swallowing of the fine grains by the larger grains in the process of grain growth [30], and this is because, in the process of grain growth, some liquid films shrink or grow at the cost of other liquid films adjacent to the grain boundary, which eventually increases the grain size significantly.

**Figure 8:**
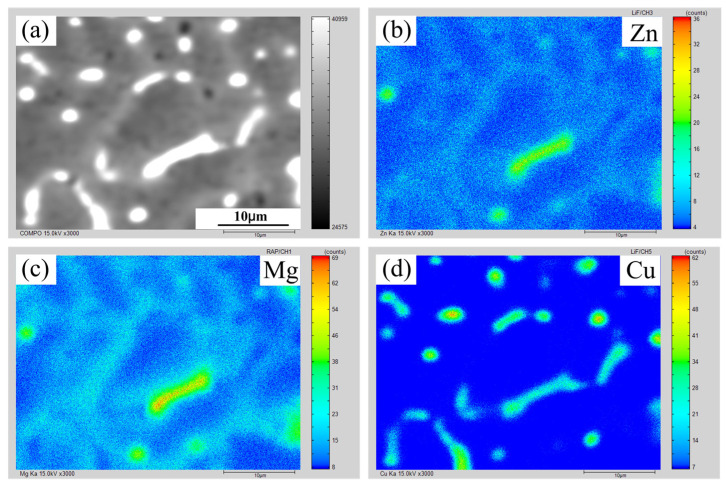
Elemental distribution of the weld under CMT+P arc mode: (**a**) secondary electron image; (**b**) distribution of Zn; (**c**) distribution of Mg; (**d**) distribution of Cu.

**Figure 9:**
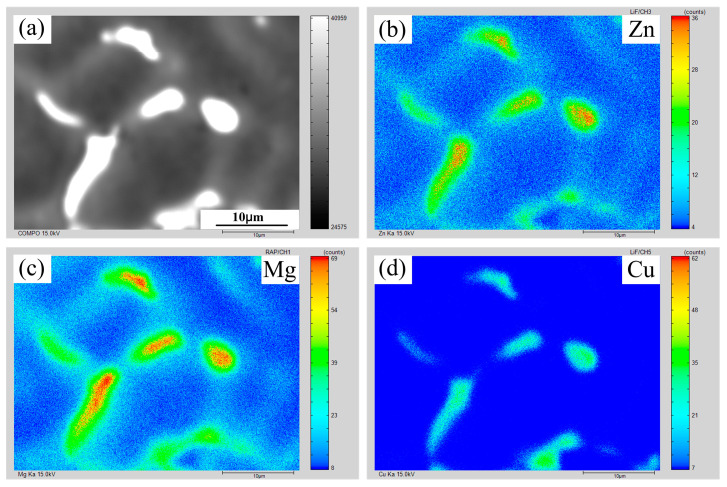
Elemental distribution of the weld under MIG arc mode: (**a**) secondary electron image; (**b**) distribution of Zn; (**c**) distribution of Mg; (**d**) distribution of Cu.

Figure 8 and Figure 9 show the EPMA mapping data of AA7075 MIG and CMT+P joints with 7055 filler wire. The micro-segregation degree of the MIG joint was higher than that of the CMT+P joint. The cooling rate of the CMT or CMT+P welding pool was lower than that of MIG, which promoted the back diffusion effect within the mushy zone, which, in turn, reduced the micro-segregation degree in the former weld slightly more than in the latter weld. The back diffusion effect reduced the solidification cracking susceptibility by decreasing the micro-segregation degree and enhancing the bridging between α-Al dendrites [31,32].

**Figure 10:**
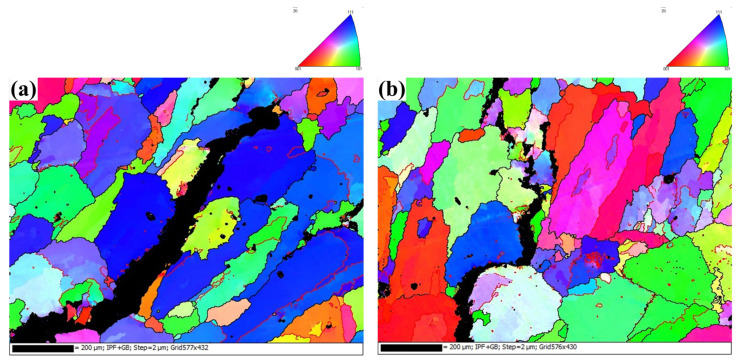
EBSD mapping of the mushy zone (7150 filler wire): (**a**) the sample welded under MIG arc mode; (**b**) the sample welded under CMT arc mode.

The microstructure of the grains within the mushy zone was further analyzed using EBSD (Figure 10). It was observed that the cracks were propagated along the grains’ boundary, a typical feature of hot cracking. The cracks within the sample welded under CMT arc mode were discontinuous, which indicated bridging between α-Al dendrites in this mode.

### 3.3. Solidification Cracking Susceptibility Analysis

The composition of both welds is shown in Table 3 (tested using the chemical titration method). It could be observed that the composition of the welds was typical of Al-Cu-Mg-Zn alloys. These data were used to calculate the *T-(f_Al_)*^1/2^ curve to reveal the solidification cracking susceptibility of each sample in this section.

**Figure 11:**
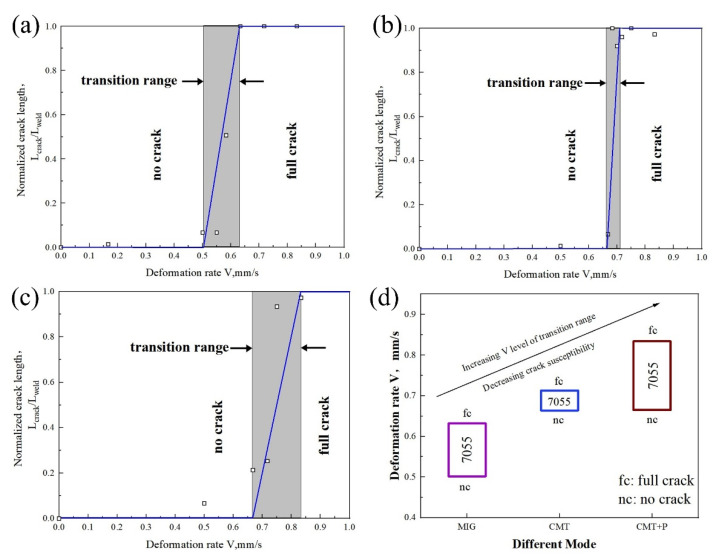
Test results of 7075 Al welded with 7055 Al as filler metal: (**a**) MIG mode; (**b**) CMT mode; (**c**) CMT+P mode; (**d**) comparison among different arc modes.

**Figure 12:**
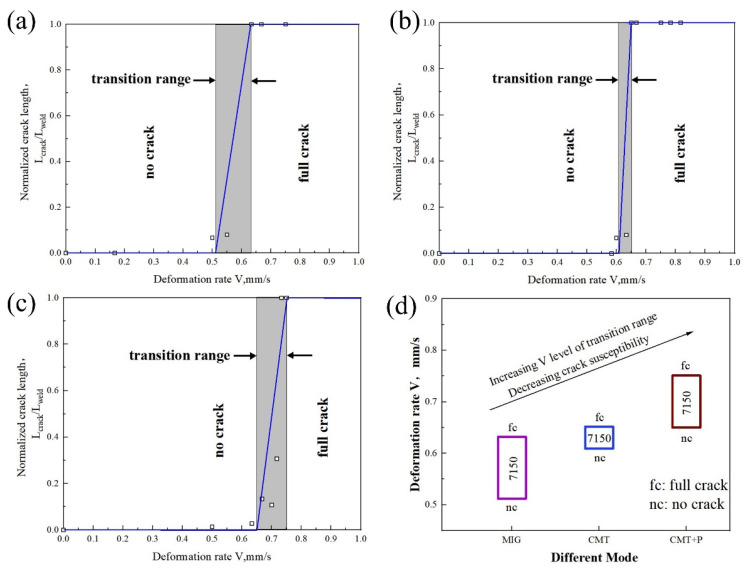
Test results of 7075 Al welded with 7150 Al as filler metal: (**a**) MIG mode; (**b**) CMT mode; (**c**) CMT+P mode; (**d**) comparison among different arc modes.

The solidification cracking susceptibility of each weld was tested using TMW (Figure 11 and Figure 12). It was observed that for the sample welded with 7055 filler wire, the critical deformation rate *Vn* followed the MIG < CMT < CMT+P sequence. The critical deformation rate *V_f_* exhibited a similar pattern. For samples welded with 7150 filler wire, the *V_n_* in MIG arc mode was significantly lower than the corresponding value in CMT and CMT+P modes. Nevertheless, the pattern of *V_f_* was similar to the alloy welded with 7055 filler wire. Generally, the critical deformation rate range for cracking shifted up in the coordinates by switching MIG mode to CMT or CMT+P arc modes, meaning that the solidification cracking susceptibility decreased.

**Figure 13:**
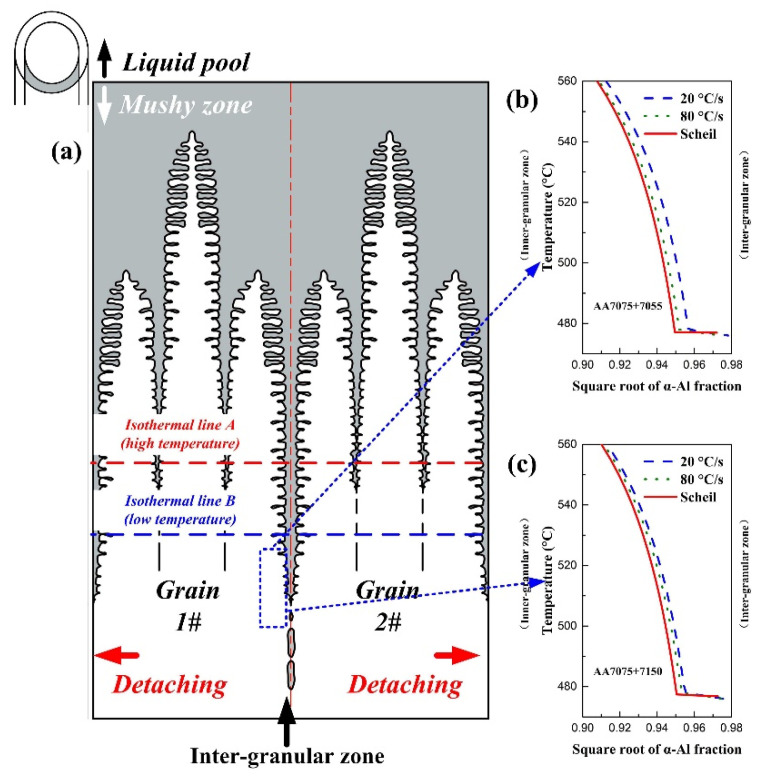
Solidification cracking susceptibility analysis: (**a**) schematic of the dendritic growth within the mushy zone; (**b**) *T-(f_Al_)^1/2^* curves of AA7075 + 7055 weld; (**c**) *T-(f_Al_)^1/2^* curves of AA7075 + 7150 weld.

The solidification cracking susceptibility reducing mechanism of the CMT-based welding method in AA7075 joints with 7055 and 7150 filler wire can be explained by Formula (1). Briefly, during the welding process, the thermal stress or strain within the weld was lower in the CMT-based welding method than in the typical MIG mode. This decreased the thermal strain factor (left-hand side in Formula (1)) by switching MIG to CMT-based arc mode. The temperature *T* vs. square root of fraction α-Al *f_Al_* curves (*T-(f_Al_)*^1/2^ curves) was then calculated (Figure 13). Importantly, the metallurgical physical meaning of *T-(f_Al_)*^1/2^ curves is presented schematically in Figure 13a. Within the mushy zone located at the liquid pool’s tail, the α-Al dendrites grew from the solid weld (cold side) toward the liquid pool’s boundary (hot side). By one-to-one correspondence operation of the isothermal line and *f_Al_*, the interface morphology near the dendrites’ boundary was approximately depicted by *T-(f_Al_)*^1/2^ curves [22]. The comparison between the *T-(f_Al_)*^1/2^ curves calculated under the Scheil and back diffusion modes revealed that decreasing the cooling rate increased *f_Al_* at the same temperature. In other words, the bridging between the adjacent α-Al dendrites was enhanced and consequently increased the value on the right-hand side in Formula (1). Thus, the solidification cracking susceptibility of the weld under CMT-based arc mode was lower than that under MIG arc mode.

**Figure 14:**
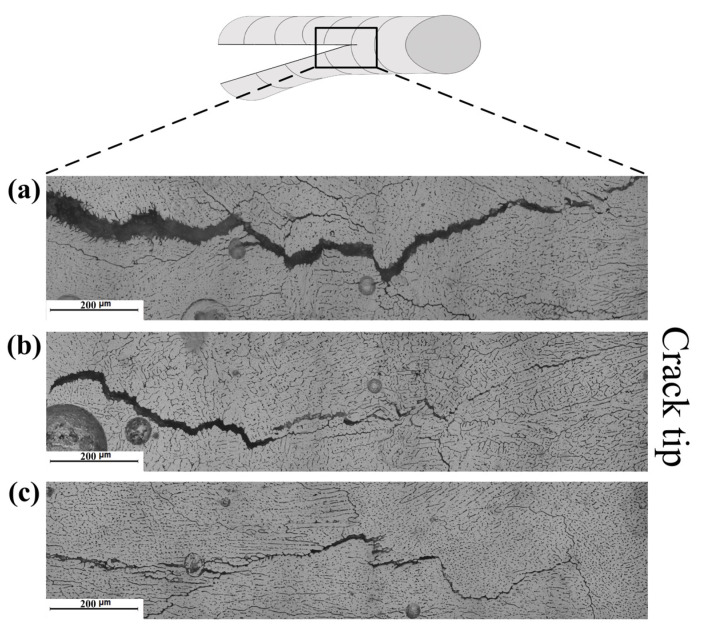
Crack tip of 7075 Al welded with 7055 Al as filler metal: (**a**) MIG mode; (**b**) CMT mode; (**c**) CMT+P mode.

**Figure 15:**
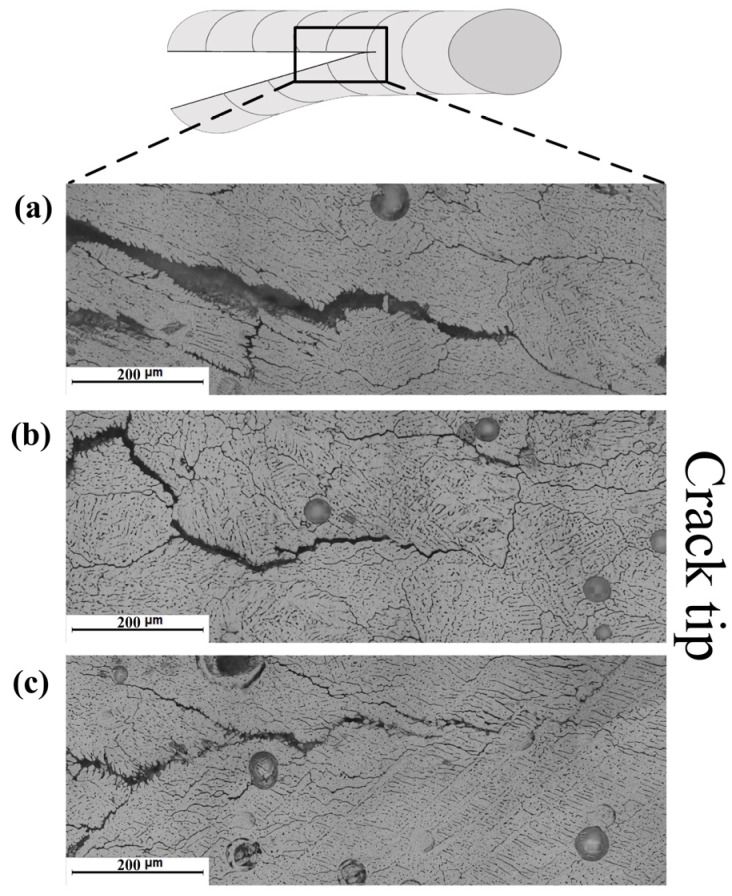
Crack tip of 7075 Al welded with 7150 Al as filler metal: (**a**) MIG mode; (**b**) CMT mode; (**c**) CMT+P mode.

Figure 14 and Figure 15 show the microstructure adjacent to the crack tip of the TMW samples under different arc modes (with 7055 filler wire). It was observed that the cracking gaps were wider in the MIG sample than in the CMT and CMT+P samples. The lower cooling rate of the welding pool in the CMT-based welding process enhanced the dendritic growth of α-Al within the mushy zone through the back diffusion effect [33]. This promoted the bridging effect between each adjacent dendrite during solidification, decreasing the solidification cracking susceptibility of the weld.

## 4. Conclusions

In this study, the solidification cracking susceptibility of aluminum alloy 7075 joints (with 7055 and 7150 filler wires) was investigated using experimental and CALPHAD methods. Three main conclusions can be drawn:

(1). The transverse-motion weldability test showed that the critical deformation rate range (from no cracking to fully cracking) shifted down in the coordinate system by switching from MIG arc mode to CMT-based arc mode during AA7075 arc welding with 7055 and 7150 filler wires. The CMT-based welding method reduced the solidification cracking susceptibility of Al-Cu-Mg-Zn alloy welds.
(2). The EPMA results for α-Al dendrites exhibited less micro-segregation in the CMT-based welded sample than in the MIG welded sample. This is because the cooling rate of the welding pool was lower in the CMT-based welding process than in the MIG welding process, which decreased the dendritic segregation through the back diffusion effect.
(3). The *T-(f_Al_)^1/2^* curve of each weld was calculated under the Scheil and back diffusion modes to analyze the effect of the weld pool’s cooling rate on the solidification cracking susceptibility. The results showed that the α-Al dendrites’ growth was promoted by the low cooling rate in the CMT-based welding process, which enhanced the bridging of adjacent dendrites within the mushy zone at the tail of the welding pool, reducing the solidification cracking susceptibility.

## Figures and Tables

**Table 1:** Compositions of base metal and filler wire (wt.%).

	Si	Cu	Mn	Mg	Cr	Zn	Ti	Zr	Al
7150 (filler wire)	<0.10	2.28	<0.10	2.32	<0.04	6.57	0.10	0.083	balance
7055 (filler wire)	<0.10	2.15	<0.05	2.08	<0.04	7.81	0.10	0.11	balance
7075 (base metal)	0.4	1.5	0.1	2.7	0.2	5.5	<0.2	-	balance

**Table 2:** Welding parameters.

	Current (A)	Voltage (V)	Welding Speed (mm/s)	CMT/P	Shielding Gas	Wire Feed Speed (m/min)
MIG	120	15.8	5	/	15 L/min	6.2
CMT	120	14.6	5	/	15 L/min	5.8
CMT+P	120	17.4	5	1:9	15 L/min	5.3

**Table 3:** Compositions of the welds.

	Si	Cu	Mn	Mg	Cr	Zn	Ti	Zr	Al
7075 + 7150	<0.10	1.88	<0.10	2.47	<0.04	5.87	0.10	0.083	balance
7075 + 7055	<0.10	1.85	<0.05	2.41	<0.04	6.46	0.10	0.11	balance

## Data Availability

Data sharing is not applicable to this article as no new data were created or analyzed in this study.

## Nomenclature

*β*	solidification shrinkage
*z*	axial direction of dendritic growth
*T*	temperature (K) or (°C)
*V*	deformation rate (mm/min)
*V_n_*	the speed when the crack began to extend (mm/min)
*V_f_*	the speed the crack fully extended (mm/min)
*f_Al_*	fraction α-Al
*f_S_*	fraction solid
*ε_local_*	local tensile strain within the mushy zone
*t*	time (s)
*v_Z_*	velocity of liquid feeding (mm/s)
*T_L_*	liquidus of AA7075
*T_S_*	solidus of AA7075
*L_crack_*	the length of the propagation crack (mm)
*L_weld_*	the length of the weld without the crack (mm)
The ratio of *L_crack_/L_weld_*	the normal length of crack propagation
